# COVID-19 Risk Perception and Adherence to Preventive Measures among Medical Students after Receiving COVID-19 Vaccination: A Multicenter Cross-Sectional Study in Egypt

**DOI:** 10.3390/vaccines11010007

**Published:** 2022-12-20

**Authors:** Abdullah Ashraf Hamad, Rasha Selim, Basma E. Amer, Rehab Adel Diab, Mahmoud Elazb, Eman H. Elbanna, MRGE Collaborative Group, Ahmed Negida

**Affiliations:** 1 Faculty of Medicine, Menoufia University, Shibin Al Kawm 32511, Egypt; 2 Medical Research Group of Egypt, Cairo 11511, Egypt; 3 Faculty of Graduate Studies and Environmental Research, Ain Shams University, Cairo 11566, Egypt; 4 Faculty of Medicine, Benha University, Benha 13518, Egypt; 5 Faculty of Medicine, Al-Azhar University, Girls Branch, Cairo 11754, Egypt; 6 Faculty of Pharmacy, Al-Azhar University, Cairo 11751, Egypt; 7 High Institute of Public Health, Alexandria University, Alexandria 21561, Egypt; 8 Faculty of Medicine, Zagazig University, Zagazig 44519, Egypt; 9 School of Pharmacy and Biomedical Sciences, University of Portsmouth, Portsmouth PO1 2DT, UK; 10 Department of Global Health, Harvard Medical School, Boston, MA 02115, USA

**Keywords:** COVID-19, vaccination, vaccines, protective measures, risk perception, medical students, Egypt

## Abstract

This study aimed to assess the perception of COVID-19 risk and the adherence to protective measures among medical students after vaccination. We conducted a cross-sectional survey on a convenience sample of students from all the 18 governmental medical schools in Egypt. A total of 2273 students participated in the online self-administered questionnaire. Around 8 in 10 (83.2%) students were fully vaccinated, of which 17.9% received the booster dose. Only 36.9% believed that COVID-19 is serious on the individual level. The majority (73.9%) strongly or slightly agreed they may become infected after vaccination if they do not follow the preventive measures. We observed a slow decline in the perceived risk of vulnerability and susceptibility to COVID-19 infection among students in parallel to a growing perception of self-efficacy and controllability. Less than one-third (28.9%) of students showed good adherence to protective measures. However, this was lower than the previously reported adherence in the same population before vaccination. Female students, those in the first academic year, those who did not contract COVID-19 infection before, and those with a higher perception of susceptibility and perceived controllability were more likely to perform better at protective measures.

## 1. Introduction

On March 2020, the World Health Organization (WHO) announced COVID-19 as a global pandemic. Soon after, several scientific guidelines confirmed on precautionary measures such as social distancing, cough etiquette, hand hygiene, and face masks as the most effective practices to reduce the viral transmission [1]. In the meanwhile, the development of the COVID-19 vaccine became a priority to control such a rapidly spreading virus. Eight months after, the first version of the COVID-19 vaccine was introduced by Pfizer-BioNTech and became available for adults older than 16 years, followed by the development, authorization, and distribution of multiple types of COVID-19 vaccines worldwide [2].

Despite the WHO recommendations for world states to vaccinate 70% of its population as soon as possible, only 57 countries have achieved this level as of May 2022 [3,4]. In the Egyptian context, and despite its efforts, Egypt hardly managed to fully vaccinate 38.48% of Egypt’s population, while only 8.26% received a booster dose by September 2020 [3].

In parallel with vaccination campaigns worldwide, reports revealed a reduction in adherence to the precautionary measures following vaccination [5,6,7,8,9]; a behavior that contributes to more viral transmission and a high likelihood of developing new strains. According to the “health belief model”, health-related behavior is influenced by individual perceptions such as perceived severity, perceived susceptibility, benefits-barriers net cost, and self-efficacy [10]. Therefore, recognizing the population’s behavior and understanding the influencing factors contributing to it are very important to guide public health policy and practice. Therefore, several studies investigated the adherence to preventive measures during the COVID-19 pandemic before and after vaccination. In response, a rapidly growing scientific literature was observed investigating population behavior during the pandemic and assessing the population’s knowledge, attitude, hesitancy, and practices.

In Egypt, several studies have assessed the Egyptian population in general and specifically the medical students’ population for their knowledge, perceptions, and attitudes regarding the COVID-19 pandemic and hesitancy against COVID-19 vaccination [11,12,13,14]. However, to the best of our knowledge, no studies have investigated the same variables following the vaccination campaign. In addition, these previous studies were limited to single or limited centers and had relatively small sample sizes.

Therefore, we conducted this study as the first in Egypt to assess medical students’ behavior toward COVID-19 preventive measures following the vaccination campaign. This study examined

(1) Current vaccination status;
(2) Adherence to protective measures;
(3) Perceptions of COVID-19 risk after the vaccination campaign.

## 2. Materials and Methods

This study was reported according to the Strengthening the reporting of observational studies in Epidemiology (STROBE) statement guidelines [15]. The study was approved by the research ethics committee of the High Institute of Public Health, Alexandria University on 28 June 2022 and was conducted according to the ethical principles of the declaration of Helsinki. Participation in this study was completely voluntary. Written consent was applied at the beginning of the questionnaire, which must be approved before answering. Confidentiality and privacy of all collected data were preserved, and the personal data were securely stored and only accessible to the statistician.

### 2.1. Study Design, Setting, and Participants

We conducted a cross-sectional study within the period from 24 May 2022 to 4 July 2022. We targeted medical students of both sexes at all the 18 governmental medical schools registered at the Egyptian Ministry of Higher Education and Scientific Research [16] (Appendix A). Students from private medical schools, students from other medical faculties such as veterinary medicine, and non-medical students were not included in this survey. Convenience sampling was employed using an online self-administered questionnaire hosted on the QuestionPro platform. The questionnaire link was shared on the official educational communication groups of all medical classes. As Egypt adopted an online approach in higher education after the COVID-19 pandemic, it was expected that all students routinely access their classes’ official groups and subsequently have equal chances to access our questionnaire.

### 2.2. Variables

The study variables were sociodemographic information and included: university, academic year, gender, age, residence, family income, COVID-19 vaccination status, and health status. Health status covered two items with self-reported yes/no questions, i.e., previous COVID-19 infection and having chronic diseases.

### 2.3. Data Sources and Measures

In addition to the sociodemographic characteristics, the online survey included two questionnaires, as follows:

1. Standard questionnaire on risk perception of an infectious disease outbreak:

This questionnaire was developed by the Municipal Public Health Service Rotterdam-Rijnmond together with the National Institute for Public Health and the Environment in the Netherlands [17]. We adopted three sections of the questionnaire that covered three aspects, i.e.,

- Perception of the seriousness of the disease (two items);
- Extent of anxiety and perception of the susceptibility to the disease (four items);
- Perceived controllability and self-efficacy of preventive measures (eight items).

The first two domains were reported on a 5-point Likert scale from strongly disagree (1 point) to strongly agree (5 points), while the third aspect was put on a 5-point Likert scale from certainly no (1 point) to certainly yes (5 points). Each domain was calculated as the sum of its items with a range of 2–10, 4–20, and 8–40, respectively. Higher scores indicate better perception.

2. Adherence to Preventive Measures:

We assembled a set of preventive measures advised by the WHO for the public becoming vaccinated (eight items) [18]. In addition, we included another six items used in previous studies on the same population [11,12]. Adherence to these measures was reported on a 5-point Likert scale from never (1 point) to always (5 points). The total adherence score was the sum of the 14 items with a range of 14–70. Students with a score ≥49 (70% of total score) were considered adherent to protective measures. We conducted a reliability analysis, which showed a Cronbach’s α = 0.865, ensuring that the questionnaire was reliable in this population.

### 2.4. Pilot

The questionnaire was piloted in a sample of 52 medical students to ensure the feasibility, clarity, and full understanding of the questionnaire. We obtained feedback from the pilot participants by asking a structured feedback question (n = 46) or direct in-person interviews (n = 6). A copy of the entire questionnaire is available as (Supplementary File S1).

### 2.5. Bias

- We estimated that the minimum completion time of the questionnaire was 150 s, so any response with less completion time was regarded as a careless response and, therefore, was excluded.
- Duplication of responses was prevented using the IP address.
- Data of the pilot study were excluded from the final analysis reported in this manuscript.

### 2.6. Sample Size and Power Calculation

Collectively, the 18 governmental medical schools in Egypt have about 83,775 students enrolled in different school years [16]. The sample size was calculated to detect an adherence rate of 28.1%, as reported in the literature [11]. To detect this adherence rate with a 95% confidence level and a 2.5% margin of error, a minimum sample size of 1224 is required. To account for the non-random sample, a design effect of 1.5 was applied. Finally, a minimum sample of 1836 students were included in this study. The sample size was calculated using EpiInfo 7.2.3.0 software according to the methods of Fahim and Negida [19].

### 2.7. Statistical Methods

Analysis was performed using Statistical Package for Social Sciences (SPSS), version 26. Data normality was tested and data were nonnormally distributed; therefore, median and interquartile ranges were used for continuous variables, whereas the number and percentage were used for categorical variables. Mann–Whitney and Kruskal–Wallis tests were used for the univariate analysis. A multiple linear regression with stepwise method (α_in_ = 0.05, α_out_ = 0.10) was performed to test the determinant factors affecting the protective behaviors. Multiple linear regression was conducted with the protective behavior score as a dependent variable and the variables that showed statistical significance in the univariate analysis and the three domains of risk perception as independent variables. A *p* value of <0.05 was considered statistically significant.

## 3. Results

We obtained complete responses from 2273 medical students. We excluded 389 responses that met the definition of careless responses. Finally, a total of 1884 responses were eligible for inclusion in the final analysis.

### 3.1. Sociodemographic Characteristics

Among 1884 students, 65.6% were in the first three academic years with a median age of 21 (19–22). Females represented 65.1% of responses. More than 8 in 10 (83.2%) students were fully vaccinated, among which 17.9% received the booster dose. More than half of students (66.6%) live in urban environments. A recognized percent of students (43.6%) reported previous COVID-19 infections, while 144 (7.6%) have chronic diseases. Family income data showed that half of the students (52.6%) consider themselves to have enough family income to save from it (Table 1).

### 3.2. Risk Perception toward COVID-19

#### 3.2.1. Perception of Seriousness

The results showed that only 36.9% of students strongly agreed that COVID-19 is serious on the individual level and can affect their life, while 61.8% strongly agreed that COVID-19 is serious on the community level and restricts the normal flow of life. The median score of perception of seriousness was 9 (7–10). Students who have chronic diseases had a better seriousness perception than other students (*p* = 0.002), while those who reported previous COVID-19 infection had less seriousness perception than their peers (*p* = 0.001) (Table 2).

#### 3.2.2. The Extent of Anxiety and Perception of Susceptibility

Nearly half of students (45.9%) were neutral about their chance of contracting COVID-19 infection in the coming year. The majority of students (73.9%) strongly or slightly agreed that even after vaccination, they can become infected if they do not follow preventive measures, while 67.4% strongly or slightly agreed that without vaccination, there is a higher risk of contracting the infection. Only 4 in 10 students (40.3%) strongly or slightly agreed that they are currently concerned about COVID-19. The median score for the extent of anxiety and perception of susceptibility score was 14 (12–16).

#### 3.2.3. Perceived Controllability and Self-Efficacy of Preventive Measures

The majority of students certainly agreed that different protective measures help to prevent infection. About half of the students (54.4%) certainly agreed that they can wear masks if it is advised, while only one third (34.8) certainly agreed that they can apply a quarantine if it is advised. The median score of perceived controllability and self-efficacy of preventive measures was 35 (32–38). There was no difference regarding study level, vaccination status, or previous COVID-19 infection (*p* > 0.05) (Table 2).

### 3.3. Protective Behaviors

#### 3.3.1. Protective Behavior Following Vaccination

For protective behavior, only 26% of students always or often kept a safe distance from others when going out, while less than 2 in 10 students (16.4%) always wore masks when going out (Table 3). The median score of protective behavior was 43 (36–50). Only 545 (28.9%) students were considered adherent to protective measures. Universities showed different scores (*p* < 0.001). However, all universities’ scores were less than 49 (70% of the total score).

#### 3.3.2. The Factors That Influence COVID-19 Protective Behaviors

As shown in Table 1, our univariate analysis indicated that females were more adherent to protective measures (*p* < 0.001). Fully vaccinated students who received the booster dose showed a better protective behavior than others (*p* = 0.043).

The multivariate analysis showed that five determinant factors, i.e., gender, academic year, reporting previous COVID-19 infection, the extent of anxiety and perception of susceptibility, and the perceived controllability and self-efficacy of preventive measures accounted for 17.8% of the variation in determining the protective behavior level (Table 4).

## 4. Discussion

### 4.1. Key Results

This study showed that half of the students felt they had an equal chance to contract COVID-19 infection. Surprisingly, students recorded a higher susceptibility to contract the infection after vaccination than the perceived risk if they had not been vaccinated. Perception of seriousness revealed that students perceived COVID-19 as 1.5 times more serious on the community level compared to perceived seriousness among individuals. The results showed a significantly higher perception of seriousness and susceptibility among females, rural residents, and students in clinical years. In addition, those who have been infected before have a lower perception of seriousness with a significant difference and become less adherent but with no significance. The result suggested that female students, those in the first academic year, and those who did not contract COVID-19 infection before were more likely to perform better at protective measures.

Despite their good perception of self-efficacy, students recorded a low willingness to apply social destination measures, and this was reflected in their low adherence to protective measures and high engagement in risky social behaviors. The results suggested that perception of susceptibility and perceived controllability would positively predict the protective behaviors.

### 4.2. Significance of the Study

This study expands the literature by providing new data on the adherence rates to COVID-19 public health measures and risk perception among Egyptian medical students after vaccination campaigns. This information is important to guide health policy makers and public health campaigns. The study showed lower adherence rates and less risk perception compared to previous reports from the same population. Therefore, more health education tools are necessary to encourage the population to adhere to public health measures and to provide accurate risk assessments of the COVID-19 situation.

### 4.3. Agreement and Disagreement with Previous Studies

Concerning risk perception, our results are highly consistent with a relatively recent study on medical students by Rayani in Iran where half of the students perceived a lower perception of susceptibility [20].

The low adherence to preventive measurements in our study is consistent with Ahmed’s study on male medical Egyptian students at Al-Azhar University in 2021 [11]. Similarly, most recent studies by Corea on the Italian population and Qin on Chinese medical students following vaccination recorded a reduction in adherence to protective behavior, mainly with risky social activities [5,8]. This is inconsistent with the high adherence rate reported by Soltan’s in Egypt, Taghrir in Iran, and Baniyas in the AUE in 2020 [12,21,22].

With regard to sociodemographic characteristics influencing risk perception, our findings are highly consistent with Abdelrahman in Qatar in 2022 and Sondakh in Indonesia in 2022, who found that females are more highly engaged in protective behavior than males [23,24]. In the same context, our results are in line with Kasemy’s study on the Egyptian community in 2020, who found that participants from rural settings showed higher protective practices rather than urban residents [25].

Our results are not in line with Qin’s study that found that a lower self-perception of susceptibility has a positive impact on protective behavior [5].

### 4.4. Strength Points and Limitations

This study is one of the earliest works investigating populations’ behaviors and protective measurements after vaccination, thus understanding how far vaccination can impact behavior patterns either for vaccinated or not-yet-vaccinated groups. Our study also has the following characteristics:

- Multicenter national study;
- Large sample size;
- Conducted after vaccination;
- Conducted on medical students;
- Validated measure (2 questionnaires).

This study targeted medical students in only governmental universities with the exclusion of private universities. We also did not include students from other medical faculties such as veterinary medicine, dentistry, physical therapy, and pharmacy, or students from non-medical schools.

### 4.5. Interpretation (Implications for Public Health)

A higher perceived seriousness on the community level reflects students’ strong concern about the epidemic burden of the disease such as the high transmission rate and further economic and social cost in comparison to the low perceived severity of the infection manifestation, especially among those who had been infected before.

High perceived susceptibility to infection even after vaccination may be due to the students’ low trust in vaccine efficacy, a factor that had been recorded by Saied et al. in Egypt who revealed that 46% of medical students had vaccination hesitancy [13]. Another explanation was given by Wong who stated that vaccinated people have a higher dread risk of infection [26].

The low adherence to protective measures in this study could be explained by “pandemic fatigue”, a phenomenon that was recently raised by the WHO. It refers to a kind of community strain due to the long exposure to stressful restricted conditions such as pandemics and leads to a reduction in the ability and motivation of the population to comply with protective behaviors, and accordingly impacts personal and community adherence [27]. In addition, the high vaccination coverage in our sample may urge students to return to their normal social life as they perceive they are protected by the vaccine. This is in line with Okamoto’s recent study on the change in student and academic staff attitudes following vaccination [28]. This behavior has been recently highlighted by the Independent Scientific Advisory Group for Emergencies who refer to it as “risk compensation”, which occurs usually when people feel they are no longer suspected to be infected, due to vaccination [29].

### 4.6. Generalizability

The results of this study can be generalized to medical students worldwide. However, the study might have limited generalizability due to the convenience sampling methods. The authors attempted to compensate for the non-random sample by increasing the sample size (design effect 1.5). However, future studies might draw a random sample from the population. Because medical students are more involved in health issues, the perception rates reported in this study might be exaggerated and, therefore, their results cannot be generalized to the whole population.

## 5. Conclusions

Our study observed that female students, those in the first academic year, and those who did not contract COVID-19 infection before were more likely to perform better at protective measures. This suggests the further attention to males and students at the clinical years in the upcoming educational campaigns. This study concluded that following the successful vaccination campaign among medical students, a slow decline in perceived risk of vulnerability and susceptibility to COVID-19 infection among students was recognized. This is in parallel with growing perceptions of self-efficacy and controllability. This slow but significant change in students’ risk perception is highly reflected in a general sharp decline in adherence to protective measurements, mainly risky social engagement.

Such ongoing and progressive coping strategies to the pandemic threat need further in-depth research to understand the change in the pattern of population behavior, which will accordingly allow policymakers to develop more effective communication strategies and further implement long-term, more adaptive interventions.

## Figures and Tables

**Table 1:** Sociodemographic profiles and analysis of protective behaviors following vaccination.

Variable	N (%)	Adherence Score Median (IQR)	Mean Rank	*p* Value
**Study level**				
Academic (1st and 2nd)	862 (45.8)	42 (36–49)	934.34	0.55 #
Clinical (3rd-internship year)	1022 (54.2)	43 (37–50)	949.38	
**Gender**				
Males	658 (34.9)	42 (35–48)	863.61	<0.001 *
Females	1228 (65.1)	43 (37–51)	984.84	
**Vaccination status**				
Full vaccinated with booster	281 (14.9)	45 (38–51)	1010.50	0.043 *
Full vaccinated without booster	1287 (68.3)	42 (36–49)	919.47	
Partially vaccinated	176 (9.3)	43 (37–50)	989.13	
Not vaccinated at all	140 (7.4)	43 (37–50)	959.08	
**Family income**				
Enough and save	991 (52.6)	43 (36–50)	937.12	0.904 *
Just enough	798 (42.4)	43 (37–50)	949.54	
Not enough	68 (3.6)	42.5 (37.25–51)	959.76	
Not enough and in debts	27 (1.4)	42 (32–50)	888.50	
**Residence**				
Big city	850 (45.1)	43 (36–50)	914.09	0.091 *
Urban center	405 (21.5)	43 (37–50)	940.38	
Rural center	215 (11.4)	42.5 (37.25–51)	1008.60	
Village	414 (22.0)	43 (37–51)	968.58	
**Reporting previous COVID-19 infection**				
Yes	821 (43.6)	42 (36–94)	915.57	0.059 #
No	1063 (56.4)	43 (37–50)	963.30	
**Having any chronic diseases**				
Yes	144 (7.6)	43 (37–52)	977.78	0.418 #
No	1740 (92.4)	43 (36–50)	939.58	

* Kruskal–Wallis H; # Mann–Whitney.

**Table 2:** Analysis for risk perception aspects following vaccination.

Variable	Perception of Seriousness of COVID-19 Score Median (IQR)	Mean Rank	*p* Value	Anxiety and Perception of Susceptibility of Infection Score MEDIAN (IQR)	Mean Rank	*p* Value	Perceived Controllability and Self-Efficacy of Preventive Measures Score MEDIAN (IQR)	Mean Rank	*p* Value
**Study level**									
Academic (1st and 2nd)	9 (7–10)	947.98	0.680 #	14 (12–16)	872.78	<0.001 #	35 (31–38)	922.33	0.138 #
Clinical (3rd- internship year)	9 (7–10)	937.88		14 (13–16)	1001.31		35 (2–38)	959.52	
**Gender**									
Males	9 (7–10)	899.48	0.01 #	14 (12–16)	889.49	0.002 #	34 (31–38)	884.2	0.001 #
Females	9 (7–10)	965.59		14 (12–16)	970.95		35 (32–38)	973.79	
**Vaccination status**									
Full vaccinated with booster	9 (8–10)	961.58	0.91 *	14 (12–16)	976.81	0.004 *	35 (32–38)	918.7	0.813 *
Full vaccinated without booster	9 (7–10)	941.37		14 (12–16)	959.02		35 (32–38)	945.27	
Partially vaccinated	9 (7–10)	930.48		14 (12–16)	863.94		35 (31–38.75)	938.22	
Not vaccinated at all	9 (7–10)	929.74		13 (12–15)	820.53		35 (32–38)	970.17	
**Family income**									
Enough and save	9 (7–10)	943.33	0.059 *	14 (12–16)	943.92	0.962 *	35 (32–38)	953.81	0.801 *
Just enough	9 (7–10)	927.65		14 (12–16)	941.33		35 (32–38)	928.27	
Not enough	9 (7.25–10)	1006.99		14 (12–16)	955.71		34.5 (32–38)	939.52	
Not enough and in debts	9 (9–10)	1188.41		14 (11–16)	891.65		34 (32–38)	955.44	
**Residence**									
Big city	9 (7–10)	931.75	0.152 *	14 (12–16)	935.1	0.623 *	34 (31–38)	892.31	0.001 *
Urban center	9 (7–10)	907.05		14 (12–16)	929.43		35 (32–38)	964.86	
Rural center	9 (8–10)	977.95		14 (12–16)	937.07		35 (32–38)	960.09	
Village	9 (7–10)	980.85		14 (12–16)	973.31		36 (32–39)	1014.53	
**Reporting previous COVID-19 infection**									
Yes	9 (7–10)	896.97	0.001 #	14 (12–16)	969.55	0.056 #	35 (32–38)	925.34	0.227 #
No	9 (8–10)	977.677		14 (12–16)	921.61		35 (32–38)	955.75	
**Having any chronic diseases**									
Yes	9 (8–10)	1076.62	0.002 #	15 (13–17)	1088.59	0.001 #	35 (32–38)	994.06	0.235 #
No	9 (7–10)	931.4		14 (12–16)	930.41		35 (32–38)	938.23	

* Kruskal–Wallis H; # Mann–Whitney.

**Table 3:** Status of protective behavior following vaccination.

Items	Answers N (%)				
	Always	Often	Sometimes	Seldom	Never
Keep a safe distance from others when going out.	134 (7.1)	357 (18.9)	638 (33.9)	425 (22.6)	330 (17.5)
Ensure good ventilation, such as by opening windows when you are indoors with others.	896 (47.6)	525 (27.9)	334 (17.7)	84 (4.5)	45 2.4)
Avoid shaking hands with others.	79 (4.2)	218 (11.6)	420 (22.3)	499 (26.5)	668 (35.5)
Avoid hugging and kissing cheeks.	205 (10.9)	334 (17.7)	468 (24.8)	412 (21.9)	465 (24.7)
Wear a well-fitting mask when going out.	309 (16.4)	376 (20)	482 (25.6)	388 (20.6)	329 (17.5)
Wash hands frequently with soap for 20 s.	708 (37.6)	554 (29.4)	381 (20.2)	146 (7.7)	95 (5.0)
Use antiseptics.	305 (16.2)	433 (23)	504 (26.8)	290 (15.4)	352 (18.7)
Avoid crowds such as malls and markets.	173 (9.2)	335 (17.8)	565 (30.0)	429 (22.8)	382 (20.3)
Avoid social meetings with relatives and friends.	68 (3.6)	179 (9.5)	353 (18.7)	495 (26.3)	789 (41.9)
Avoid social events like marriage or parties.	110 (5.8)	198 (10.5)	366 (19.4)	478 (25.4)	732 (38.9)
Cover any sneeze in your bent elbow.	1098 (58.3)	387 (20.5)	213 (11.3)	87 (4.6)	99 (5.3)
Stay at home when feeling flu-like symptoms.	454 (24.1)	543 (28.8)	552 (29.3)	216 (11.5)	119 (6.3)
Isolate yourself at home if you get in contact with COVID-19 infected patients.	658 (34.9)	448 (23.8)	425 (22.6)	208 (11.0)	145 (7.7)
Eat healthy food, get enough sleep and exercise regularly.	250 (13.3)	492 (26.1)	662 (35.1)	321 (17.0)	159 (8.4)

**Table 4:** Multivariate linear regression analysis on the protective behaviors following the vaccination.

Variables	Partial Regression Coefficient	Standard Error	Standardized Partial Regression Coefficient		t	*p* Value	95% CI
Constant	8.396	1.891	-	4.441	<0.001	(4.689, 12.104)	
Gender	1.516	0.452	0.071	3.355	0.001	(0.630, 2.402)	
Academic year	−0.320	0.132	−0.053	−2.431	0.015	(−0.579, −0.062)	
reporting previous COVID-19 infection	0.957	0.432	0.047	2.214	0.027	(0.109, 1.805)	
the extent of anxiety and perception of susceptibility	0.771	0.081	0.210	9.524	<0.001	(0.612, 0.930)	
the perceived controllability and self-efficacy of preventive measures	0.615	0.045	0.298	13.645	<0.001	(0.526, 0.703)	

R^2^ = 0.178, F = 81.511, *p* < 0.001. CI confidence interval.

## Data Availability

The data presented in this study are available on request from the corresponding authors.

## Supplementary Materials

The following supporting information can be downloaded at: https://www.mdpi.com/article/10.3390/vaccines11010007/s1, A copy of the entire questionnaire is available as Supplementary File S1.

## Appendix A

Medical schools included in our study

1. Faculty of medicine, Ain Shams university;
2. Faculty of medicine, Alexandria university;
3. Faculty of medicine, Assiut university;
4. Faculty of medicine, Benha university;
5. Faculty of medicine, Beni Suef university;
6. Faculty of medicine, Cairo university;
7. Faculty of medicine, Fayoum university;
8. Faculty of medicine, Helwan university;
9. Faculty of medicine, Kafr Elsheikh;
10. Faculty of medicine, Mansoura university;
11. Faculty of medicine, Menia university;
12. Faculty of medicine, Menoufia university;
13. Faculty of medicine, Port Said university;
14. Faculty of medicine, Sohag university;
15. Faculty of medicine, South Valley university;
16. Faculty of medicine, Suez Canal university;
17. Faculty of medicine, Tanta university;
18. Faculty of medicine, Zagazig university.
