# Supplementary material for: COVID-19 Risk Perception and Adherence to Preventive Measures among Medical Students after Receiving COVID-19 Vaccination: A Multicenter Cross-Sectional Study in Egypt
Source: Vaccines (Basel). 2022 Dec 20;11(1):7. doi: 10.3390/vaccines11010007 (PMC9866727; doi:10.3390/vaccines11010007)
Supplement: Supplementary file 1 [file vaccines-11-00007-s001.zip › vaccines-1984075-supplementary.pdf]

The questionnaire

COVID-19 risk perception and adherence to preventive measures following vaccination among medical students: A cross-sectional study in Egypt

:Hello
You are invited to participate in our survey regarding Covid-19. In this survey, medical students will be asked to complete a survey that asks questions about risk perception and adherence to preventive measures. It will .take approximately 3 minutes to complete the questionnaire
.Completing this questionnaire means you agree to participate in this study
Your participation in this study is completely voluntary. There are no foreseeable risks associated with this project. However, if you feel uncomfortable answering any questions, you can withdraw from the survey at any .point
Your survey response will be strictly confidential and data from this research will be reported only in the .aggregate. Your information will be coded and will remain confidential
If you have questions at any time about the survey or the procedures, you may contact **Hamad** by email .abdullah.hamad744@gmail.com Thank you very much for your time and support
.Please start with the survey now by clicking on the **START** button below

Socio-demographic section

University
1. Cairo
2. Ain Shams
3. Alex
4. Mansoura
5. Helwan
6. Assiut
7. South valley
8. Menia
9. Sohag
10. Beni Suef
11. Zagazig
12. Tanta
13. Menoufia
14. Benha
15. Kafr El Sheikh
16. Fayoum
17. Suez canal
18. Port Said

Academic year
1. 1st
2. 2nd
3. 3rd
4. 4th
5. 5th
6. 6th
7. Internship

Sex
1. Male
2. Female

:Age

Residence
1. Big City
2. Urban center
3. Rural center
4. Village

Family income/economic level
1. Enough and save
2. Just enough
3. not enough
4. not enough and in debts

Vaccination status
1. not vaccinated at all
2. Partially vaccinated (one dose)
3. Full vaccination without booster (two doses)
4. Full Vaccination with booster (three doses)

:Health status

	yes	no
Get infected with Covid-19 before	❏	❏
Have any chronic disease	❏	❏

smoking status
.1 Smoker
.2 Ex-smoker
.3 Never smoked

:Risk perception section

.Perception of seriousness and susceptibility

	Strongly agree	Slightly agree	Neutral	Slightly disagree	Strongly disagree
On the individual level, COVID-19 infection is serious (Serious: complicated and affect your life)	❑	❑	❑	❑	❑
On the community level, COVID-19 infection is serious (Serious: Affect many people and restrict normal flow of social life)	❑	❑	❑	❑	❑
.You have a big chance of getting COVID-19 infection in the coming year	❑	❑	❑	❑	❑
After vaccination, you can get COVID-19 infection if you don't follow preventive measures	❑	❑	❑	❑	❑
in case you have not been vaccinated against COVID-19 or the vaccine was not available, you would be at higher risk of infection in the coming year	❑	❑	❑	❑	❑
Currently you are concerned about COVID-19	❑	❑	❑	❑	❑

:Perception of efficacy and self-efficacy

?Do you think that the following measurements help to prevent COVID-19 infection

	Certainly yes	Probably yes	Perhaps yes-perhaps no	Probably not	Certainly not
?Frequent hand hygiene	❑	❑	❑	❑	❑
?Wearing masks	❑	❑	❑	❑	❑
?social distancing	❑	❑	❑	❑	❑
?Quarantine	❑	❑	❑	❑	❑

Intention to carry out the measures

?Do you think that you can apply the following measurement if this is advised

	Certainly yes	Probably yes	Perhaps yes-perhaps no	Probably not	Certainly not
Hand hygiene	❑	❑	❑	❑	❑
Wearing mask	❑	❑	❑	❑	❑
social distancing	❑	❑	❑	❑	❑
quarantine	❑	❑	❑	❑	❑

Adherence to preventive measures section

:Please choose what accurately describes your behavior now

After vaccination

	Always	Often	Sometimes	Seldom	Never
Currently, Do you keep a safe distance from ?others when going out	❑	❑	❑	❑	❑
Currently, do you avoid shaking hands with ?others	❑	❑	❑	❑	❑
Currently, do you avoid hugging and kissing ?cheeks	❑	❑	❑	❑	❑
Currently, do you wear a well-fitting mask ?when going out	❑	❑	❑	❑	❑
Currently, do you wash your hands frequently ?with soap for 20 sec	❑	❑	❑	❑	❑
?Currently, do you use antiseptics	❑	❑	❑	❑	❑
Currently, do you avoid crowds such as malls ?and markets	❑	❑	❑	❑	❑
Currently, do you avoid social meetings with ?relatives and friends	❑	❑	❑	❑	❑
Currently, do you avoid social events like ?marriage or parties	❑	❑	❑	❑	❑
Currently, do you cover any cough or sneeze in ?your bent elbow	❑	❑	❑	❑	❑
Currently, do you stay at home when feeling ?flu-like symptoms	❑	❑	❑	❑	❑
Currently, do you eat healthy food, get enough ?sleep and Exercise regularly	❑	❑	❑	❑	❑
Currently, if you get in contact with covid-19 infected patients would you isolate yourself at ?home	❑	❑	❑	❑	❑
Currently, do you ensure good ventilation, such as by opening a window, when you are indoors ? with others	❑	❑	❑	❑	❑
